# Predictive Modeling of Liquid Density and Surface Tension for Sustainable Aviation Fuels Using Nuclear Magnetic Resonance Atom Types

**DOI:** 10.1021/acs.energyfuels.4c05601

**Published:** 2025-02-10

**Authors:** Robert P. Parker, Mark Kelly, Tiarnán Watson-Murphy, Mohammad Reza Ghaani, Stephen Dooley

**Affiliations:** Trinity College Dublin, College Green, Dublin 2 D02 PN40, Ireland

## Abstract

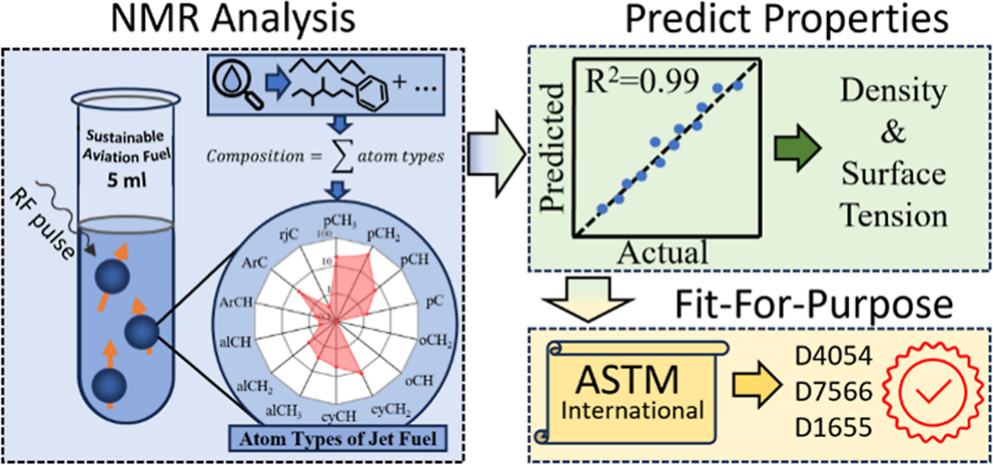

Prescreening of sustainable aviation fuels (SAFs) is crucial for early stage development and ASTM D4054 evaluation. This study develops models to predict two key properties: temperature-dependent liquid density and surface tension of complex hydrocarbon mixtures. ^1^H ^13^C heteronuclear single quantum coherence nuclear magnetic resonance spectroscopy is used to determine atom type compositions. Multiple linear regression models, trained on 1241 liquid density and 1260 surface tension experimental data points, identified seven key atom types and a temperature-dependent term as predictors. Applied to fossil-derived and synthetic fuels, density predictions had an error range of 0.00–5.35%, and surface tension predictions ranged from 0.29–4.41%. The prescreening method proved to be effective for predicting critical fuel properties in early stage SAF development.

## 1 Introduction

Sustainable aviation fuels (SAFs) or, more formally, synthetic aviation turbine fuels, produced using carbon neutral feedstocks, are important in mitigating the environmental impact of aviation, reducing greenhouse emissions, and enabling long-term sustainability in the industry. Introducing new aviation fuels and fuel additives in accordance with ASTM D4054^1^ poses challenges for fuel producers due to the number of fuel property measurements and the high volume of fuel required by the evaluation process, presenting a high economic barrier to entry and a deterrent for investment.

Prescreening is a novel technique in which the properties of the SAF candidates can be predicted using statistical regression or other mathematical models based on some form of compositional information. Heyne et al.^2^ have summarized six key properties to which prescreening should first be applied to aid in eventual fuel qualification. These include density at 15 °C and surface tension at 22 °C. Density, the ratio of mass to volume, is a critical property that can be used as an indicator for many other properties such as specific energy, viscosity, heat of combustion, and atomization.^2^

Surface tension is the energy required to increase the surface area of a liquid. Cohesive forces hold molecules together in a drop while adhesive forces arise between the liquid and the interface; these both influence surface tension and give compounds their unique property.^3^ Surface tension crucially affects the droplet size and atomization dynamics of fuels in gas turbine combustors, in turn impacting the operability and efficiency of the engine.^2^ As SAFs are intended to serve as “drop-in” replacements for conventional fuels, ASTM D4054^1^ and ASTM D7566^4^ outline specific fuel properties and acceptable range limits that a SAF must exhibit if the fuel is to be approved for use with aviation original equipment manufacturer airframes and engines. Both surface tension and density are of primary importance in this context and are, therefore, central to the focus of the current study.

In the context of SAF evaluation and approval according to ASTM D4054, prescreening methods that require minimal fuel quantities are advantageous as they reduce costs while providing sufficient data for accurate property predictions. Heteronuclear single quantum coherence (HSQC) nuclear magnetic resonance (NMR) spectroscopy is a promising technique for such applications due to its nondestructive nature and capability to analyze complex mixtures.

NMR spectroscopy probes molecular structures by utilizing the magnetic properties of specific atomic nuclei. For hydrocarbon fuels, traditional NMR is limited by overlapping peaks which impacts the ability to assign atom types, and HSQC-NMR offers enhancements by correlating the chemical shifts of protons (^1^H) with those of heteronuclei, such as carbon (^13^C) or nitrogen (^15^N), enabling detailed structural analysis. This method is highly effective for identifying and quantifying atom types, where each atom type is defined by its unique electronic environment within the molecular structure.

For fuel analysis, ^1^H ^13^C HSQC-NMR offers several distinct advantages that are complementary to chromatographic and infrared spectroscopy methods. While certain gas chromatography detectors are capable of subppm detection sensitivity, ^1^H ^13^C HSQC-NMR achieves similar sensitivity without requiring extensive sample preparation, analyte derivatization, or calibration, which is often necessary in gas chromatography analyses. Infrared spectroscopy excels at identifying molecular degrees of freedom, usually associated with molecular vibrations, which can, in principle, be attributed to the presence of specific functional groups. However, the wavelengths at which vibrational modes are accessed may overlap between the vibrations of different discrete functional groups, making infrared spectroscopy challenging for highly complex mixtures, such as fuels. While NMR can also have shared resonances, this occurs to a lesser extent, and it provides quantitative compositional insights, making it uniquely suited for the detailed analysis of hydrocarbon mixtures. Additionally, ^1^H ^13^C HSQC-NMR allows for in situ analysis and provides valuable structural information about molecular interactions within complex mixtures. Compared to gas chromatography and infrared spectroscopy, NMR spectroscopy generally involves higher initial costs due to instrument acquisition and maintenance, although the cost of an experiment is less than 10 euros.

In this study, ^1^H ^13^C HSQC-NMR is employed to quantify atom types in hydrocarbon aviation turbine fuel-like mixtures, offering a detailed breakdown of the mole fraction of each atom type. This information is of utility in understanding the composition and properties of the fuel. The ability of ^1^H ^13^C HSQC-NMR to handle the inherent complexity of hydrocarbon mixtures, while also providing both compositional and structural insights, makes it a valuable tool in fuel research and development.

Liquid density and surface tension predictive models for small samples of aviation fuel using an array of analytical techniques are available in literature.^5−11^ Yang et al.^11^ used GCxGC to determine the hydrocarbon group-type composition of SAF, relying on group elution patterns based on volatility and polarity. Monte Carlo sampling was used to bound the range of properties of SAF. This method reported a coefficient of determination (*R*^2^) of 0.93 for density at 15 °C and a *R*^2^ of 0.64 for surface tension at 22 °C for 20 predicted SAF values. Heyne et al.^6^ demonstrated that incorporating vacuum ultraviolet identification into GCxGC analysis reduced the uncertainty in various fuel property predictions, with confidence intervals reduced up to 21 times compared to GCxGC with flame ionization detection, though *R*^2^ values were not reported. Wang et al.^10^ used FTIR to characterize the average fuel structure in terms of functional groups and used linear methods to estimate hydrocarbon fuel properties. This method reported an *R*^2^ of 0.83 for density at 15 °C and an *R*^2^ of 0.85 for surface tension at 22 °C.

Group contribution methods are techniques that estimate the properties of pure components through the summation of contributions from specific molecular groups or structures within the compound.^12−15^ Predictive models for density that utilize group contribution methods already exist in the literature.^16−21^ Skander and Chitour^19^ conducted a study which used molecular structures and a correction term based on the relative structure location to CH_3_ groups and carbon atom placement in ring structures for the estimation of physical properties of hydrocarbons, including liquid density at 20 °C. The resulting model predicted liquid density of all hydrocarbon classes with an average absolute relative deviation (AARD) of 1.3%. Wakeham et al.^21^ utilized carbon atoms/bonds and molecular energies within hydrocarbons to estimate the liquid density and critical properties with an average relative error (ARE) of 1.17% for liquid density at 20 °C.

Similarly, group contribution methods for the estimation of surface tension are available in the literature.^17,22−27^ Gharagheizi et al.^23^ conducted a study where the surface tension for ionic liquids was computed based on their chemical structures. These structures were defined by the anions and cations within the ionic liquids. After evaluating 34 different chemical structures, they found that only 19 were effective for accurately estimating the surface tension. The model’s performance was characterized by an AARD of 3.20%. This study of Gharagheizi et al.^23^ represents the first instance in literature where surface tension estimation of a liquid relied solely on group contributions and temperature.

Building upon this approach, Shahsavari et al.^27^ predicted the surface tension of ionic liquids using a group contribution method based on the amount of each constituent element present, rather than chemical structures. The resulting model displayed an AARD of 4.16%. These studies demonstrate that estimating surface tension through group contributions is feasible; however, its application is limited to known samples with discrete quantifiable chemical structures.

While group contribution methods utilizing atom types, as proposed in this study, have been previously employed in the literature,^28,29^ their application to the prediction of properties such as liquid density and surface tension in complex fuel-like mixtures remains unexplored. In this regard, we have previously developed the ^1^H ^13^C HSQC-NMR methodology for the purpose of atom type analysis of fuels.^28−31^ It has been shown that this methodology can identify and precisely quantify discrete atom types by integrating peaks on the ^1^H spectrum and assigning with assistance from the HSQC spectrum to identify the atom types.^31^

In this study, multilinear regression (MLR) models, based on atom type group contributions, are developed for liquid density and surface tension for the specific use case of aviation fuel complex mixtures.

## 2 Methodology

The basis of the methodology is the assumption that the surface tension and density of fuels are principally dependent on the molecular structure, which can be measured by NMR, and that this relationship can be learned by mathematical analysis of an appropriate database of molecular structures for each property (i.e., in this study, temperature-dependent liquid density and surface tension).

### 2.1 Atom Types by ^1^H ^13^C HSQC-NMR Spectroscopy

**Figure 1:**
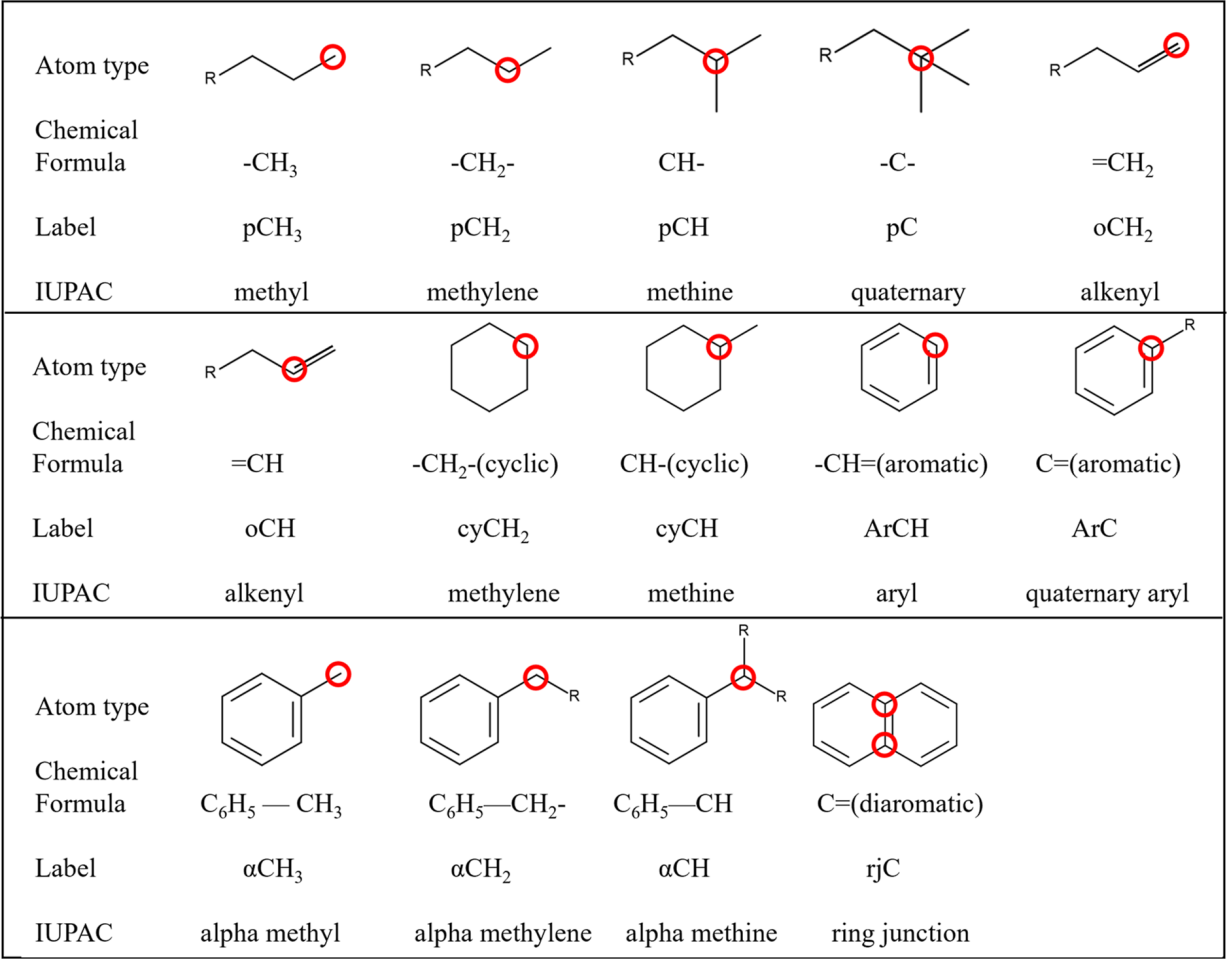
Jet aviation fuel atom type formula classification and structure, with shorthand nomenclature.^31,32^

The atom types utilized in this study are listed in Figure 1. The method of Ure et al.^31^ has been adapted to additionally enable the identification and quantification of aromatic-ring junction carbon atom types.^32^ As introduced by Ure et al.^31^ and further developed in this work, ^1^H ^13^C HSQC-NMR identifies the unique nuclear and electronic environments associated with each atomic type within hydrocarbon molecules that constitute typical liquid transportation fuels. This identification inherently includes the geometrical configurations of each atomic type and molecular group ensemble.

**Figure 2:**
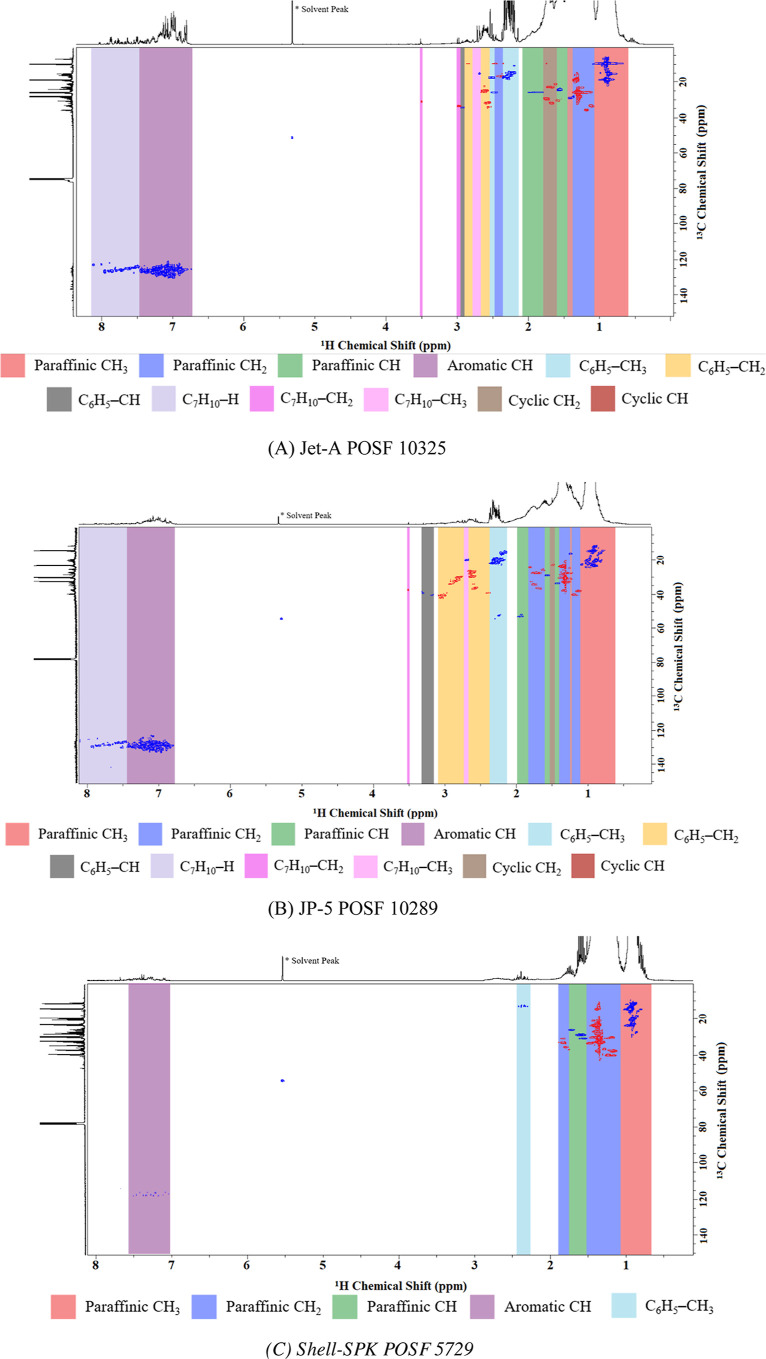

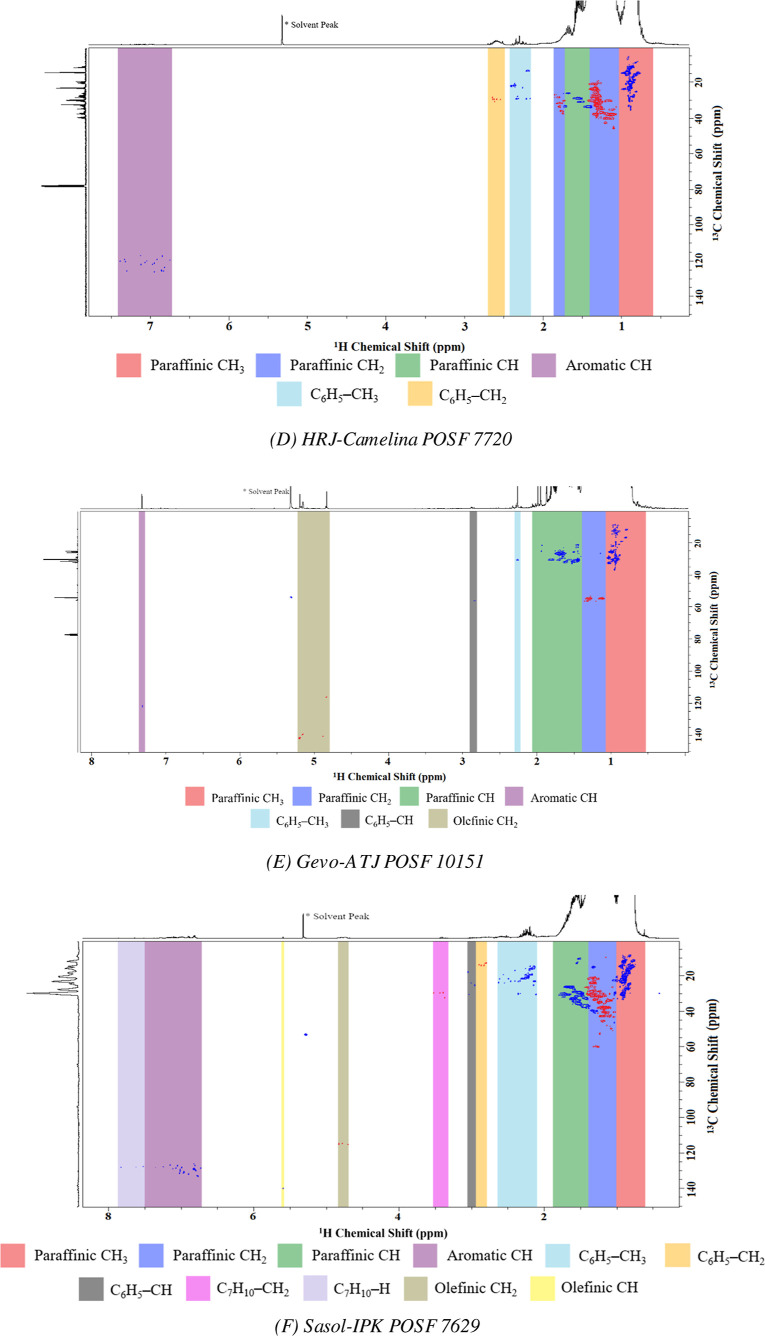
^1^H ^13^C HSQC-NMR spectra for 2 fossil fuels (Jet A, JP5) and 4 synthetic aviation fuels (Shell-SPK, HRJ-Camelina, Gevo-ATJ, Sasol-IPK), which are used to assign atom types. These fuels are used to validate the models presented.

**Figure 3:**
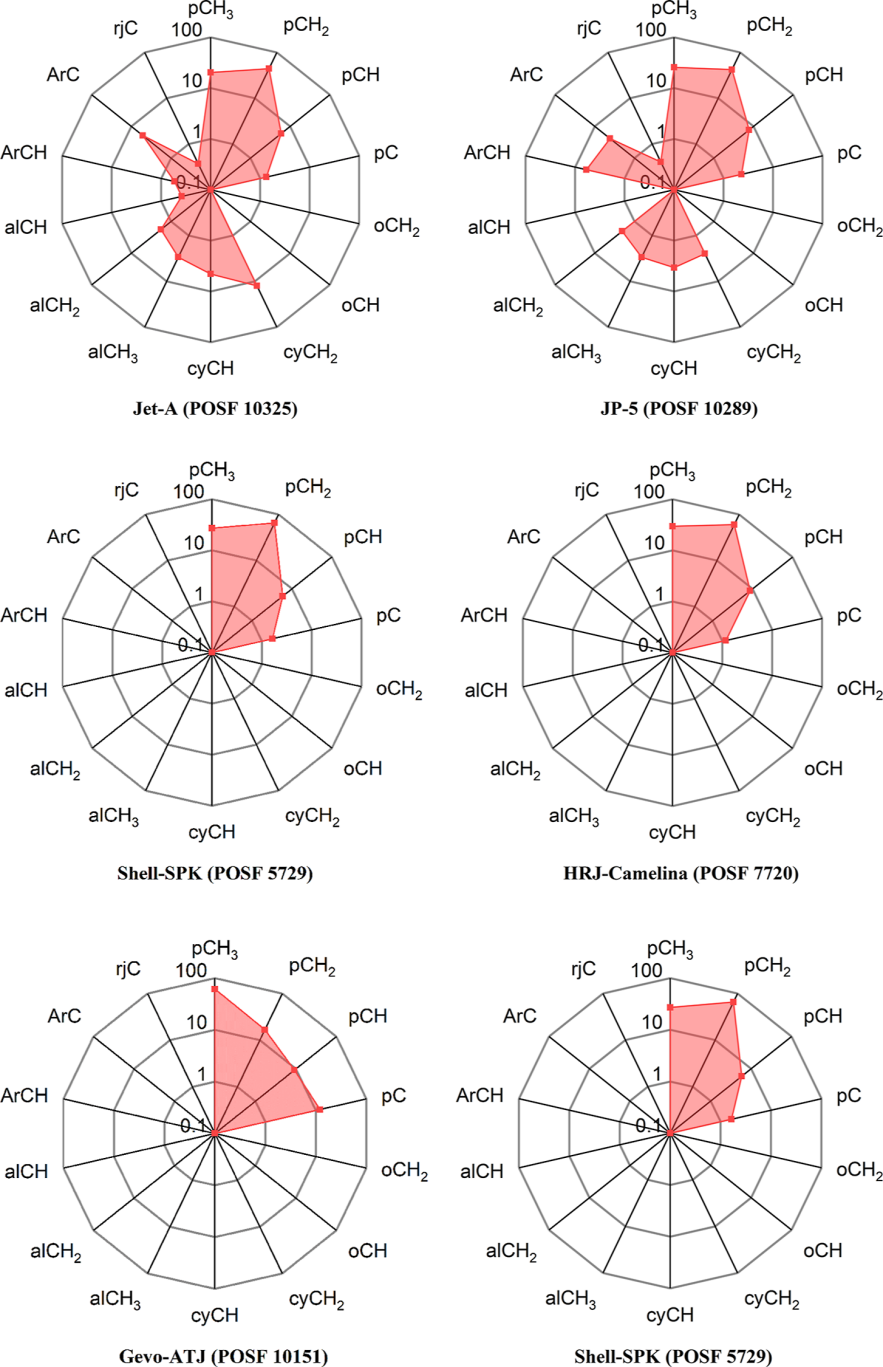
^1^H ^13^C HSQC-NMR atom type mole fraction on a log-radar plot for fossil aviation fuels and synthetic aviation fuels, showing considerable compositional differences. The axis represents the mass fraction of each atom type in the sample.

To validate the full procedure, a set of six real fuels with unknown atom type compositions were analyzed. The ^1^H ^13^C HSQC NMR spectra for these real fuels are presented in Figure 2, and the untruncated ^1^H spectra as well as the data of each ^1^H spectrum are provided in Supporting Information (S1, S5) and the atom type distribution in Figure 3, as they were quantified to be used as validation data for the models presented. HSQC, quantitative ^1^H, and qualitative ^13^C NMR spectra were collected using a Bruker AVANCE III 400 NMR spectrometer with the parameters suggested by Ure et al.^31^ HSQC spectroscopy produces a two-dimensional NMR spectrum wherein the ^1^H and ^13^C spectra are on the *x*- and *y*-axes, respectively. The intersection of data from these two axes, termed a cross-peak, represents a specific C–H bond. Carbon atoms with an odd number of bonded hydrogen atoms (i.e., paraffinic CH and CH_3_) have an opposite phase to carbon atoms with an even number of bonded hydrogen atoms (i.e., paraffinic CH_2_). Hence, a phase editing procedure is applied to the data to allow for the quantification and display of both sets of data on one spectrum. As the magnitude of the ^1^H chemical shift is due to the local bonding environment of the nuclei, it is possible to assign regions of the *x*-axis (^1^H spectrum) to each atom type. The ^1^H ^13^C HSQC spectrum was used to assign the spectral regions for each atom type for each sample. These regions were then integrated in the quantitative ^1^H spectrum to determine the atom type composition in mol % hydrogen. The spectrum was then used to calculate the inferred mol % carbon composition using the extended method of Watson-Murphy et al.,^32^ which introduces a calculation for identifying ring junction carbon atom types. The uncertainty in the quantification of atom type mole fractions has been investigated separately.^32^ Four model aviation turbine fuels of known composition and increasing complexity from 5 to 12 components were used. Across 14 atom types, Watson-Murphy et al. found maximum and minimum absolute errors of 0.94 and 0.00 mol % carbon, respectively, indicating an impressive accuracy of routine ^1^H ^13^C HSQC-NMR analysis. Watson-Murphy et al. further indicated that the small imprecisions noted are principally due to signal-to-noise arising from the short scan times, which can be readily improved by increasing acquisition time.

The preparation of solutions and the choice of solvents are crucial factors in NMR analysis. Solvents must be carefully selected to avoid spectral interference, and the sample-to-solvent ratio must be optimized to ensure clear and accurate atom type quantification. In this study, a 1:4 ratio of sample to deuterated solvent (0.2 g of sample to 0.8 g of deuterated dichloromethane) was used, which is an adaptation of the methodology presented by Ure et al.^31^ This ratio was found to balance resolution and intensity, avoiding issues such as decreased peak intensity from excessive solvent or peak overlap from too much sample. The choice of deuterated dichloromethane was specifically made to minimize interference with the atom type distribution observed in jet fuels. The H/C ratio of each fuel, measured using an Exeter Analytical CE 440 Elemental Analyzer, is used to deduce the quantity of quaternary paraffinic carbon present, which does not appear on the spectra due to having no bonded hydrogens. Although the methodology employed accounts for all atom types described, it does not consider trace impurities that may be present in the fuel. These impurities are described in ASTM D4054^1^ to be in a ppm unit basis. However, only organic impurities (water, carbonyls, alcohols, esters, and phenols) that are directly bonded to carbon or hydrogen atoms would be detectable in the NMR spectra used in this analysis. These are not identifiable through the methodology employed and would be included in the integration for atom types if they are within the range.

Additionally, this methodology was applied to real aviation and gasoline fuels available in our laboratory: POSF 10325 Jet-A and POSF 10289 JP-5 are both fossil derived aviation fuels, POSF 5729 Shell Fischer–Tropsch Synthetic Paraffinic Kerosene derived from natural gas,^33^ POSF 7720 Hydrotreated Esters and Fatty Acid (HEFA) derived from Camelina oil,^33^ POSF 10151 Gevo Alcohol-to-Jet (ATJ) derived from iso-butanol, and POSF 7629 Sasol-IPK derived from coal.^33^Figure 2 shows the assigned ^1^H ^13^C HSQC spectrum for each of these fuels, with the atom type assignment presented in Table 1.

**Table 1:** Operating Equation Declaring Learned Atom Type Coefficients and Associated *p*-Values for Temperature-Dependent Liquid Density and Surface Tension Models

		α_0_	α_1_	α_2_	α_3_	α_4_	α_5_	α_6_	α_7_	α_8_
density, ρ [kg/m^3^]	coefficient	1101	–0.9	–556.9	539.2	995.8	–69.9	430.1	28.3	771.4
	*p*-value	<0.01	<0.01	<0.01	<0.01	<0.01	<0.01	<0.01	<0.01	<0.01
surface tension, σ [mN/m]	coefficient	61.9	–0.1	–36.7	30.2	58.2	–6.3	14.4	–2.8	49.9
	*p*-value	<0.01	<0.01	<0.01	<0.01	<0.01	<0.01	<0.01	<0.01	<0.01
equation	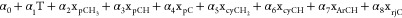									

### 2.2 Data, Regression, and Models

There are three technical conditions that must be met when employing MLR for this purpose: (1) the atom types that most significantly contribute to surface tension and density must be identifiable through the ^1^H ^13^C HSQC-NMR spectrum of the real fuel requiring analysis, (2) there must be property data for pure components and/or hydrocarbon mixtures of sufficient quality and quantity to train the numerical model, and (3) the resulting operating equation must be concise and only contain statistically significant parameters. This approach enhances our understanding of the importance of, and interactions between, atom types, the molecules they comprise, and the molecular properties they define.

To train the MLR model, density and surface tension data of experimental measurements were gathered by a critical examination of the suitable literature^34−54^ for both pure components and their mixtures. It contains 2501 experimental data points, generally comprised of normal paraffins, iso-paraffins, cycloparaffins, aromatics, and binary mixtures of these classes; the statistical descriptors (minimum, maximum, mean, and standard deviation) for surface tension, density, temperature, and each atom type in the training database are available in Supporting Information (S7). The distribution in the temperature space shows that most experimental measurements are reported between 20 and 30 °C (see Supporting Information S2). Surface tension and density can be assumed, based on experimental measurements, to have a linear relationship with temperature over specific ranges (see Supporting Information S3). For this purpose, experimental data measured at temperatures at least 50 °C below the boiling point of each component were included in the data set to ensure sufficient temperature diversity and consistency with the single linear temperature-dependent term in the model. For model generation, a discrete database is utilized for each property. Each experimental value in the database is associated with an atom type composition, as depicted in Figure 1. The atom type compositions for each component are provided in Supporting Information (S6). Each component was then linked to the corresponding temperature at which liquid density or surface tension measurements were taken, as sourced from the literature.^34−54^ MLR models were produced with operating equations of the form
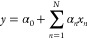
1where α_0_ is the intercept, α_*n*_ are the coefficients learned for each independent variable, *x*_*n*_ are the atom type mole fractions or temperature, and the dependent variable, and *y* is liquid density or surface tension. Individual models for temperature dependent liquid density and temperature-dependent surface tension were then trained using the Linear Regression module from the SciKit Learn Python package.^55^

**Figure 4:**
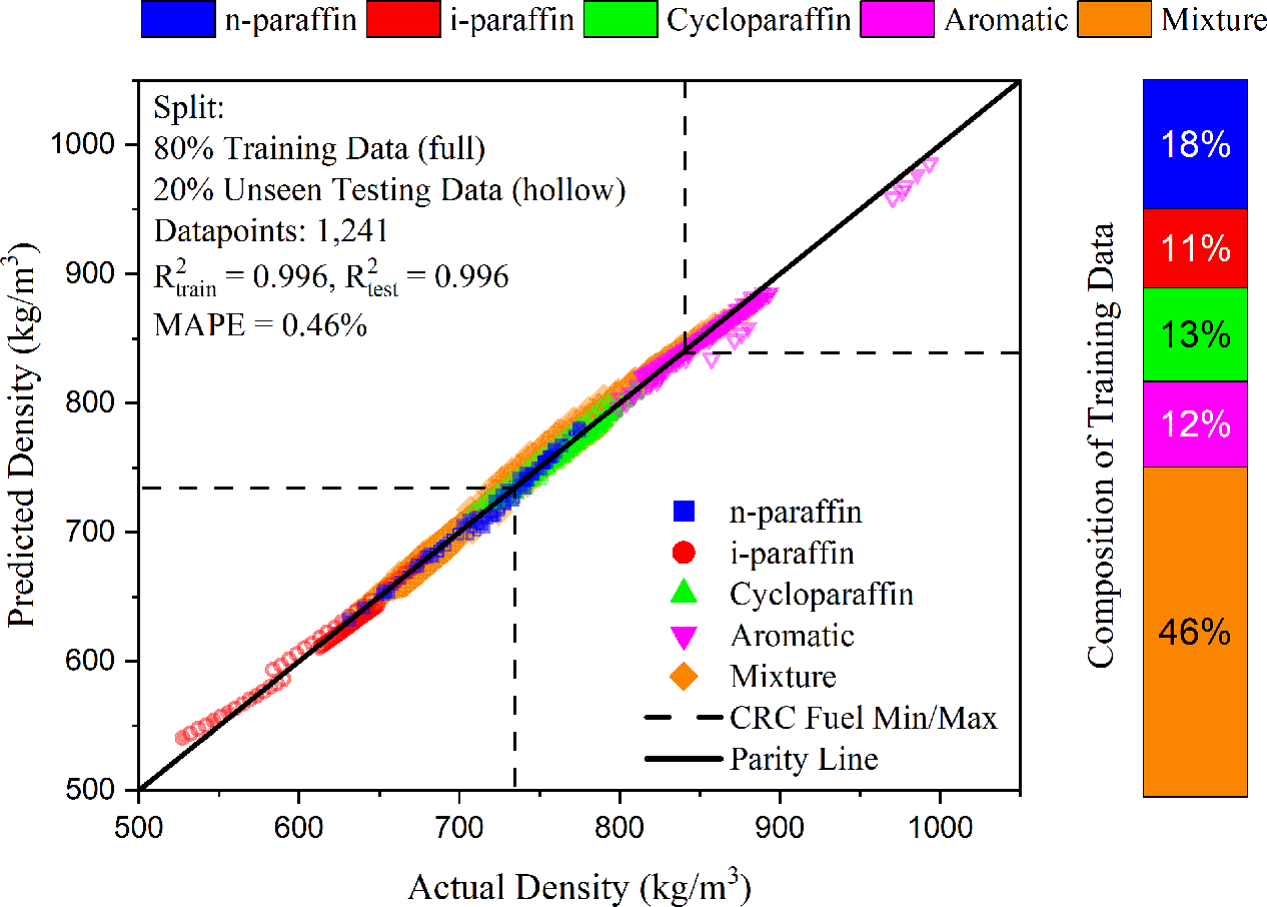
Temperature-dependent density, predicted density versus actual measured density with an 80:20 split in training (seen data, hollow symbols), and testing (unseen data, full symbols) data.

**Figure 5:**
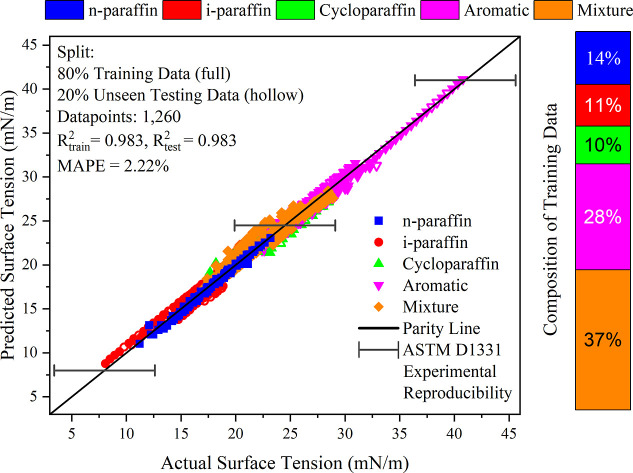
Temperature-dependent surface tension, predicted surface tension versus actual surface tension with an 80:20 split in training (unseen data, hollow symbols), and testing (seen data, full symbols) data. The error bars represent the reproducibility standard deviation for Material C (du Noüy) as per ASTM D1331,^58^ indicating potential experimental uncertainties in actual surface tensions.

The density data set with 1241 data points and the surface tension data set with 1260 data points were each divided into five random 80:20 splits for training and testing, using the average values for the model statistics. The training data sets are used to train the MLR models, and the testing data sets validate the performance of the models, ensuring that they have been reliably trained. The independent variables chosen to describe each property were systematically evaluated using the *p*-value of each coefficient. Each model was developed using the backward selection methodology, where independent variables with a *p*-value greater than 0.05 were individually removed, and the model was retrained with fewer atom types until the *R*^2^ degraded significantly (<0.95). To further enhance model stability and prevent overfitting, ridge regression was applied. This method extends conventional linear regression by adding a regularization step within the method applied prior to implementing gradient descent, which penalizes excessive coefficient magnitudes, thus preventing coefficient inflation and improving model robustness. A range of ridge regularization parameters were tested using a grid search method, and the parameter that minimized validation error was selected. This approach ensures an optimal bias-variance trade-off, prevents coefficient inflation, and maintains predictive accuracy across data sets. Only variables with statistically confirmed significant influence are retained, resulting in more concise and reliable predictive models. The models presented in Figures 4 and 5 and Table 1 satisfy the criteria regarding *p*-values while achieving the highest coefficient of determination among all models generated.

### 2.3 Model Validation

True validation data, i.e., density or surface tension measurements for real or even realistic (e.g., model fuels, surrogates) complex liquid fuels, are sparse. A further challenge in demonstrating validation arises from the novelty of the atom type NMR methodology; however, this limitation due to novelty also affects other prescreening approaches such as GCxGC.^6,11^ As a result of this, atom type assignments, particularly as obtained through the Ure et al.^31^ method of this study, are not available in the existing literature. Hence, studies of this nature are constrained by the availability of fuel samples within the laboratory, the measurement capability of the laboratory, and/or the availability of data on the property of interest from the literature. The limitations stem from the limited availability of atom type data in the existing literature, as the ^1^H ^13^C HSQC-NMR technique is novel and not yet widely adopted. Consequently, all measurements were conducted in-house to quantify atom types on the fuels available, enabling the study of density and surface tension properties in the selected fuels.

## 3 Results and Discussion

The temperature-dependent liquid density (ρ) and surface tension (σ) regression model coefficients, along with their respective *p*-values, are listed in Table 1. The first notable result is that the best-performing models for both liquid density and surface tension utilize the same set of atom types as independent variables, indicating that these atom types are fundamentally influential for both properties. Second, the *p*-values of all variables are below 0.01, underscoring the strong influence of each atom type within the predictive equations. This observation was rigorously interrogated and confirmed to be valid. Analysis of potential model equations revealed that density and surface tension exhibit a linear relationship with temperature across all pure components and mixtures, excluding regions corresponding to transition phases. Generality in temperature dependence, rather than atom type specificity, served as the basis for formulating operating equations. Coupled temperature-atom type terms were found to be statistically insignificant, whereas independent linear terms remained significant. Consequently, decoupling these effects minimizes the number of parameters, improving both simplicity and interpretability. A group contribution methodology ensures that each term explicitly accounts for the distinct mole fraction of each atom type. This approach contrasts with frameworks that combine temperature dependence with group contributions (for example, the temperature coupled contributions proposed by Elbro et al.^56^), which inherently leads to increased model complexity. The internal consistency of the training data represents the largest source of uncertainty in the models. The models cannot describe unseen data more accurately than they describe their training data; any inconsistencies within the training set ultimately define the limit of predictive performance.

The predictive performance of each model was then assessed by evaluation against two discrete databases: (1) the training and testing data set discussed previously comprised of pure components and pure component mixtures from the literature; (2) the reference real fuels available at Trinity College Dublin as outlined above.

### 3.1 Atom Type Composition of Real Fuels

To evaluate model performance against (2), atom type data are needed. The results of the ^1^H ^13^C HSQC-NMR assignment are provided in Table 2 and graphically in Figure 3, which we propose as a useful way to simplify complex fuel compositions, allowing for a visual inspection of “what a fuel looks like”. The conventional fossil aviation fuels such as Jet-A POSF 10325 and JP-5 POSF 10289 have similar atom type distributions with a large quantity of paraffinic atom types and a notable fraction of aromatic and cycloparaffinic types. The synthetic aviation fuels have a similarly large quantity of paraffinic atom types but do not contain as high levels of aromatics, and strikingly no cycloparaffins for Shell-SPK POSF 5729.

**Table 2:** ^1^H ^13^C HSQC-NMR Atom Type Compositions for Real Synthetic and Fossil Aviation Fuels^a^

	atom type composition (mol % carbon)													
sample (POSF)	pCH_3_	pCH_2_	pCH	pC	oCH_2_	oCH	cyCH_2_	cyCH	αCH_3_	αCH_2_	αCH	ArCH	ArC	rjC
Jet-A (10325)	20.34	44.08	5.97	1.33	0.00	0.00	12.54	4.54	2.97	1.79	0.39	0.54	5.14	0.37
JP-5 (10289)	23.99	42.20	8.38	2.69	0.00	0.00	2.5	3.69	2.79	2.05	0.00	6.4	4.84	0.47
Shell-SPK (5729)	27.11	65.30	5.83	1.64	0.00	0.00	0.00	0.00	0.03	0.00	0.00	0.06	0.03	0.00
HRJ-Camelina (7720)	29.64	60.30	8.71	1.17	0.00	0.00	0.00	0.00	0.03	0.01	0.00	0.09	0.05	0.00
Gevo-ATJ (10151)	58.46	17.02	10.09	14.29	0.05	0.00	0.00	0.00	0.01	0.00	0.02	0.02	0.03	0.00
Sasol-IPK (7629)	48.68	27.17	17.57	5.69	0.02	0.01	0.00	0.00	0.21	0.07	0.03	0.22	0.32	0.01

a These data are shown graphically in Figure 3.

### 3.2 Performance to Validation Data Set #1:80:20 Split Training Set

The predictive performance of the models using 80% (“seen”) training data and 20% (“unseen”) testing data is shown in Figures 4 and 5 for density and surface tension, respectively. For easier distinction, these figures are also presented separately in the Supporting Information (S4). The deviation in slope seen between 500 and 600 kg/m^3^ is due to a single low molecular weight component at varying temperature. In contrast, the data points at 950 kg/m^3^ correspond to a diaromatic compound at varying temperatures.
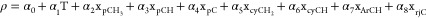

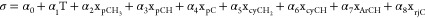


While the models reviewed in previous group contribution studies do not focus on predicting unknown samples of fuels or complex mixtures, it remains valuable to compare their results with those obtained in this work. The density model published by Skander and Chitour^19^ achieved an AARD of 1.3%, and the model by Wakeham et al.^21^ reported an ARE of 1.17%. In contrast, the density model presented in this work demonstrates superior performance with an AARD of 0.43% and an ARE of −0.006%. Similarly, earlier surface tension models by Gharagheizi et al.^23^ and Shahsavari et al.^27^ reported AARDs of 3.20% and 4.16%, respectively. In comparison, the surface tension model introduced in this study exhibits an improved AARD of 2.12%.

Table 3 presents the performance metrics for the temperature-dependent density and surface tension models, split across training and testing data sets. High *R*^2^ values for both density (0.996) and surface tension (0.983) in both sets indicate strong predictive capability and show that the models effectively capture atom type contributions without bias. Slight increases in error metrics, such as MAPE (0.43% to 0.45% for density and 2.11% to 2.22% for surface tension), are expected as the models encounter new data. The predictive errors compare favorably to standard reproducibility metrics. For density, ASTM D1298^57^ specifies reproducibility at 1.2 kg/m^3^ and repeatability at 0.5 kg/m^3^; the RMSE values of 4.23 kg/m^3^ (training) and 4.46 kg/m^3^ (testing) are higher than expected, but density measurements are very accurate. For surface tension, ASTM D1331^58^ specifies four materials for the du Noüy ring experiment with varying reproducibility and repeatability standard deviations and limits. Among these, Material C (solvent-borne alkyd paint) most closely aligns with the hydrocarbon-based nature of jet fuels, particularly because the solvents are generally composed of aromatic and aliphatic hydrocarbons. The standard deviation of reproducibility is shown in Figure 5. When compared with the surface tension model, the RMSE values of 0.60 mN/m (training) and 0.64 mN/m (testing) align well with the results of this standard.

**Table 3:** Train and Test Performance Metrics for the Temperature-Dependent Density and Surface Tension Models

	density		surface tension	
	train	test	train	test
*R*^2^	0.996	0.996	0.983	0.983
MAPE	0.43%	0.45%	2.11%	2.22%
RMSE	4.23 kg/m^3^	4.46 kg/m^3^	0.60 mN/m	0.64 mN/m
MAE	3.19 kg/m^3^	3.37 kg/m^3^	0.45 mN/m	0.47 mN/m

### 3.3 Performance to Validation Data Set #2: Reference Real Aviation Fuels

**Figure 6:**
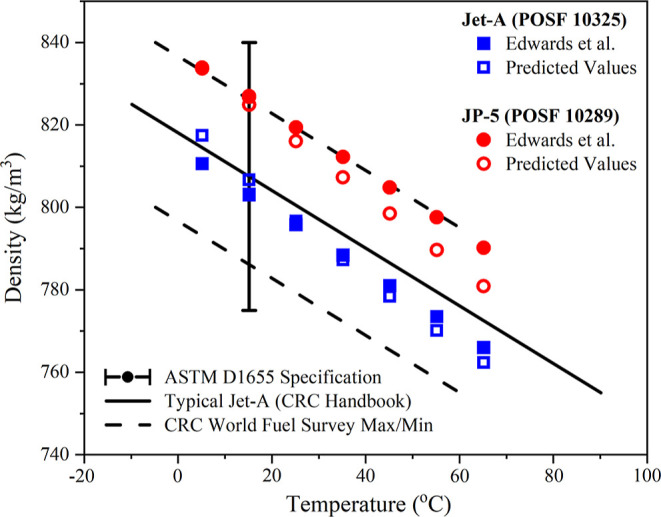
Liquid density versus temperature, literature experimental measurements^59^ (full symbols), and unseen model predictions of this study (hollow symbols). The ASTM fit-for-purpose properties are included as lines for reference. The ASTM D1655 specification is used as a qualification (limit) metric in the initial ASTM D4054 evaluation.

The performance of the density model when predicting the density of reference real aviation fuels is summarized in Table 4 and Figure 6. It is important to appreciate that for these fuels, the models have only been trained on pure components and simple mixtures, and hence, complexity in the atom type distribution seen in these fuels is yet unseen. The comparisons thus present a meaningful demonstration of the effectiveness of the entire methodology and the fundamental concepts.

**Table 4:** Performance Analysis of Machine Learned Density Model and Surface Tension Model Produced in This Work for Fossil and Synthetic Aviation Fuels at 15 °C^a^ and 16 °C^b^ for Density^59−62^ and 22 °C for Surface Tension^59^

	density			surface tension at 22 °C		
sample (POSF)	actual density [kg/m^3^]	model density [kg/m^3^]	absolute error in density [%]	actual surface tension [mN/m]	model surface tension [mN/m]	absolute error in surface tension [%]
Jet A (10325)	803^a^	805	0.21	24.8	24.9	0.29
JP-5 (10289)	826^a^	825	0.23	25.7	26.1	1.68
Gevo-ATJ (10151)	760^a^	719	5.35	n/a	19.1	n/a
Sasol-IPK (7629)	761^a^	729	4.23	n/a	20.5	n/a
Shell-SPK (5729)	737^b^	740	0.39	n/a	21.6	n/a
HRJ-Camelina (7720)	757^b^	757	0.00	n/a	22.7	n/a

The density model demonstrates a strong predictive capability for the density of the reference fuels, with an average error of 1.74% for densities at 15 and 16 °C, as shown in Table 4. The notable error for Gevo-ATJ and Sasol-IPK seems to stem from a high paraffinic quaternary carbon composition. This is something which will be examined in more detail in future work.

The predictive performance of the density model against fossil jet aviation fuels is analyzed in Figure 6. The density model is evaluated using the only temperature-dependent experimental data available, those from Edwards et al.^60^ for Jet-A POSF 10325 and JP-5 POSF 10289, showing an average error of 0.44% and a maximum error of 1.18% at 65 °C for JP-5 POSF 10289. Importantly, Figure 6 shows that the overall methodology would predict both fuels to be inside the limits presented by the ASTM D1655^63^ standard specification for aviation turbine fuels. Such a verification is the purpose of prescreening. The observed deviation in slope between measured and predicted densities may arise from limitations in the training data set’s compositional coverage, particularly for complex, multicomponent fuels, as well as the model’s linear approach, which may not fully capture nonlinear temperature dependencies.

**Figure 7:**
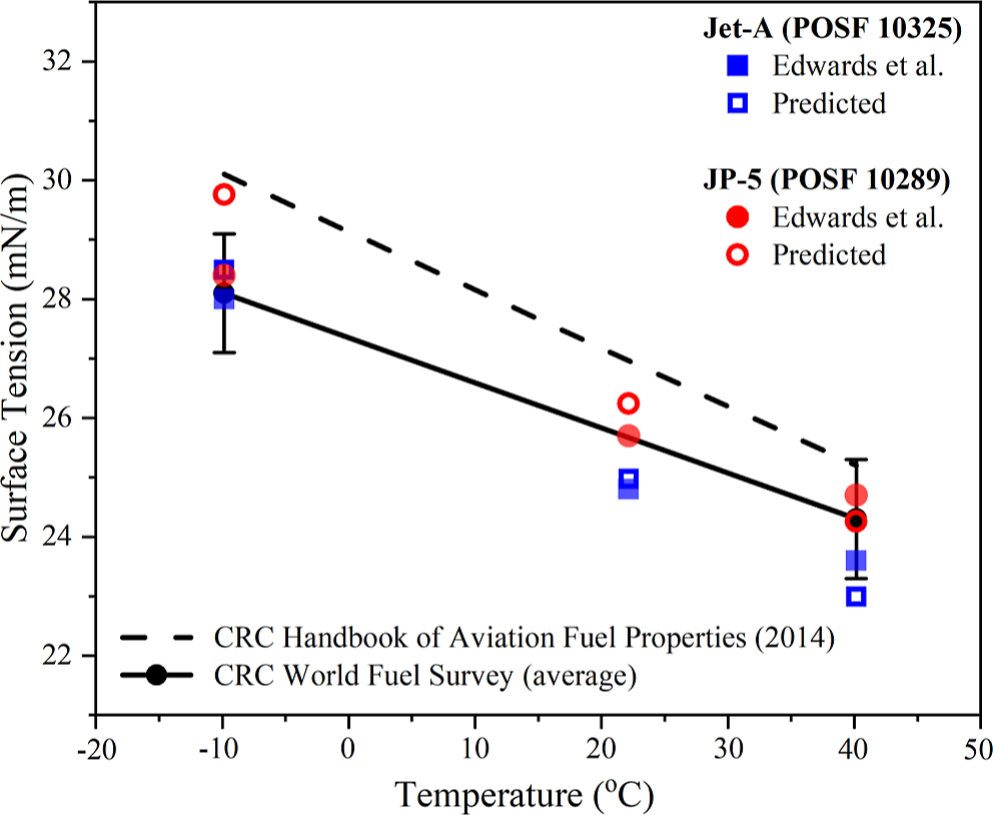
Surface tension versus temperature, literature experimental measurements^59^ (full symbols), and unseen model predictions of this study (hollow symbols). For reference, the ASTM fit-for-purpose properties are included as lines. There are no specification limits for surface tension in the ASTM D4054 evaluation.

With regard to surface tension, much less experimental data are available for true unseen testing. Of the fuels analyzed by ^1^H ^13^C HSQC-NMR, only Jet-A POSF 10325 and JP-5 POSF 10289 have measurements reported. The surface tension predictions for the other reference fuels are supplied in Table 4 and graphically in Figure 7, showing the aviation fuels to be in the range 17–22 mN/m at 22 °C. This is slightly lower than the range specified in the CRC world fuel survey and within the range to conform with ASTM D4054, standard practice for evaluation of new aviation turbine fuels and fuel additives.

Moreover, Figure 7 presents a direct comparison of the entirely unseen model, which predicted temperature-dependent surface tension to the measurements of Edwards et al. for Jet-A POSF 10325 and JP-5 POSF 10289. The model demonstrates a strong predictive capability, showing an average error of 2.3%. More specifically, an error of 0.29–2.99% for Jet-A POSF 10325 and 1.68–4.41% for JP-5 POSF 10289 demonstrates the potential of the overall technique.

Regarding the uncertainty associated with the model, future work will include an analysis with model fuels and a detailed hydrocarbon analysis of real fuels, which will allow for the quantification of error in the ^1^H integration. This approach enables sequential application of uncertainty to each atom type, providing an overall uncertainty in the predictions based on the uncertainties in the inputs. This aspect will be comprehensively addressed in future studies.

### 3.4 Compositional Space of the Training Data

Figures 6 and 7 indicate that while both models do achieve a useful predictive capability, the models do not perfectly capture the temperature-dependent density and temperature-dependent surface tension behaviors of JP-5 in particular. Though uncertainty in the experimental measurement should not be discounted as a source of this discrepancy, it is expected that a more complex training database will result in a better capture of this behavior.

The surface tension model shows less accuracy, which can be indicative of an inherently greater complexity in these phenomena compared with density (Figure 4 vs Figure 5). Despite these shortcomings, the predictions for both properties are sufficiently accurate to classify the analyzed fuels as “fit-for-purpose”, thus fulfilling the primary objective of this study.

The reference real fuels, which serve as the benchmark for evaluating the predictive ability of the models, are highly complex fluids composed of thousands of discrete molecules. This complexity highlights the need for a more diverse set of complex mixtures in the training data to capture the full range of the compositional variability. Ideally, a model would be trained on a data set that comprehensively and evenly represents the compositional space of the fluids being analyzed. However, this is challenging in practice due to the vast compositional diversity found in conventional and synthetic aviation fuels as well as the significant time and cost associated with conducting experimental determinations for reference pure component mixtures.

**Figure 8:**
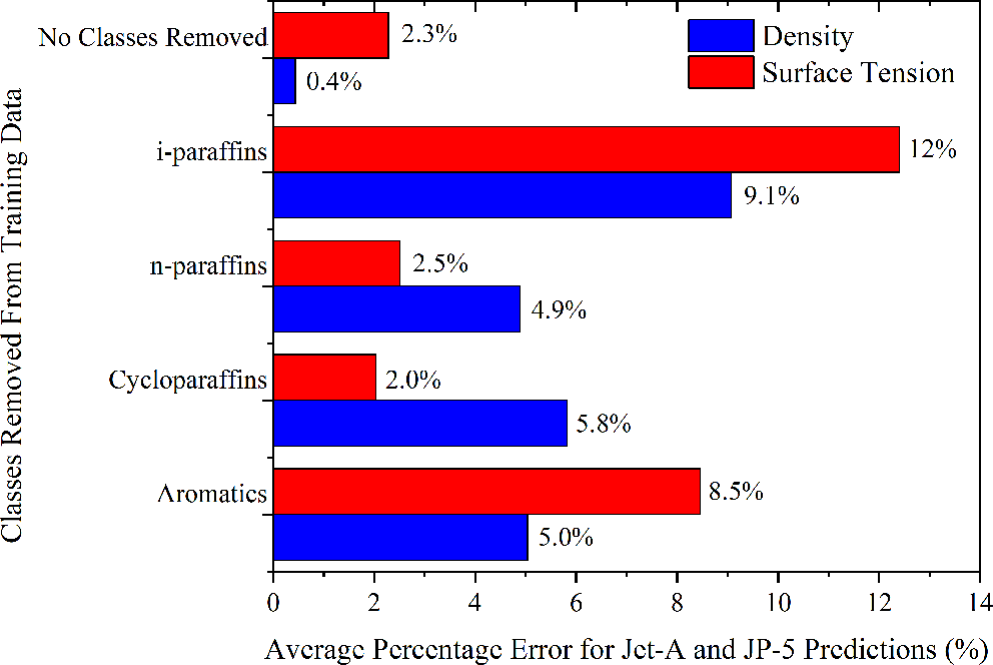
Average percentage error in the model prediction of temperature-dependent density and temperature-dependent surface tension of Jet-A POSF 10325 and JP-5 POSF 10289, relative to experiment,^59^ for different descriptions of the compositional space. The *y*-axis denotes the singular absence of each molecular class from the training data.

From Figure 3, the large variabilities in the composition space occupied by the real reference fuels become obvious, particularly the lower prevalence of aromatic and cycloparaffinic atom types in the synthetic aviation fuels. To study the effect of compositional space on model performance, the influence of this factor is analyzed using the original data sets. In this analysis, the training set comprises only pure components, as mixtures may include multiple classes. Model training by multiple linear regression was performed again, but with individual molecular classes removed from the training set and the performance of the resulting model assessed; all other variables were held constant. Figure 8 shows the results against density and surface tension data for Jet-A POSF 10325 and JP-5 POSF 10289.

In particular, for both density and surface tension, the removal of isomerized paraffins and aromatics has a considerable degrading effect on the predictive ability, emphasizing their importance.

With respect to density, the removal of any hydrocarbon class leads to a considerable, but more consistent, increase in the model error on the order of 5%. The extent of performance degradation for the surface tension model is more varied depending on which molecular class training data are removed. This feature further points to the more complex nature of the surface tension physical phenomenon over that of liquid density.

The analysis evidences the importance of having a comprehensive coverage in molecular class/atom type training data in order to accurately predict the properties of real complex fuels, by this and similar methodologies. Furthermore, and of fundamental scientific value, though density and surface tension are usually labeled as “physical” properties as no covalent bonds are broken or formed, the analysis presented here makes it clear that these phenomena are dependent on the molecular structure.

## 4 Conclusions

Predictive models for liquid density and surface tension have been created, and their applicability to prescreening of SAF candidates has been investigated. These models, produced through multiple linear regression, utilize atom type mole fractions as a predictor. Atom types are quantified by ^1^H ^13^C HSQC-NMR, which requires only milliliters of sample. Separate databases comprising 1241 data points for density and 1260 data points for surface tension measurements were compiled from the literature.

These data were used to train and test simple numerical models by which temperature-dependent density and temperature-dependent surface tension can be described on the basis of atom types. The liquid density model displays an excellent predictive performance of unseen pure components and pure component mixtures, with an average error of 0.47%. This model also predicts the density of complex liquid fuels such as real fossil and synthetic aviation fuels and gasolines to within 5.35%. The surface tension model similarly has an excellent predictive performance, showing an average error of 2.22% to unseen pure components and pure component mixtures and predicting the surface tension of real aviation fuels to within 4.41%. These results show promise for the concept of prescreening of SAF candidates; however, the predictive performance of the models can be improved by the inclusion of more complex mixtures in the training data set and improving the coverage of the training data set in compositional space, which requires considerable experimental data. Future work will focus on developing further prescreening models for critical jet fuel characteristics, such as the distillation curve, flash point, and derived cetane number, to expand the predictive framework and methodology.

## Supporting Information Available

^1^H NMR spectra for all reference fuels used, temperature distribution of density and surface tension data sets, temperature dependence of surface tension with *n*-heptane and density with Jet-A and JP-5, parity plots for training and testing data of density and surface tension (PDF)
Proton spectrum data for all reference fuels used, surface tension atom types, density atom types, and training data statistics (XLSX)
