# Supplementary material for: Predictive Modeling of Liquid Density and Surface Tension for Sustainable Aviation Fuels Using Nuclear Magnetic Resonance Atom Types
Source: Energy Fuels. 2025 Feb 10;39(7):3690–702. doi: 10.1021/acs.energyfuels.4c05601 (PMC11848819; doi:10.1021/acs.energyfuels.4c05601)
Supplement: Supplementary file 1 — ef4c05601_si_001.pdf [file ef4c05601_si_001.pdf]

Supplemental Information

Predictive Modelling of Liquid Density and Surface Tension for Sustainable Aviation Fuels Using Nuclear Magnetic Resonance Atom Types

Robert P. Parker*, Mark Kelly, Tiarnán Watson-Murphy,
Mohammad Reza Ghaani*, Stephen Dooley

Trinity College Dublin, Dublin, Ireland

S1 – Conventional and Synthetic Aviation Turbine Fuel ^{1}H Spectra:

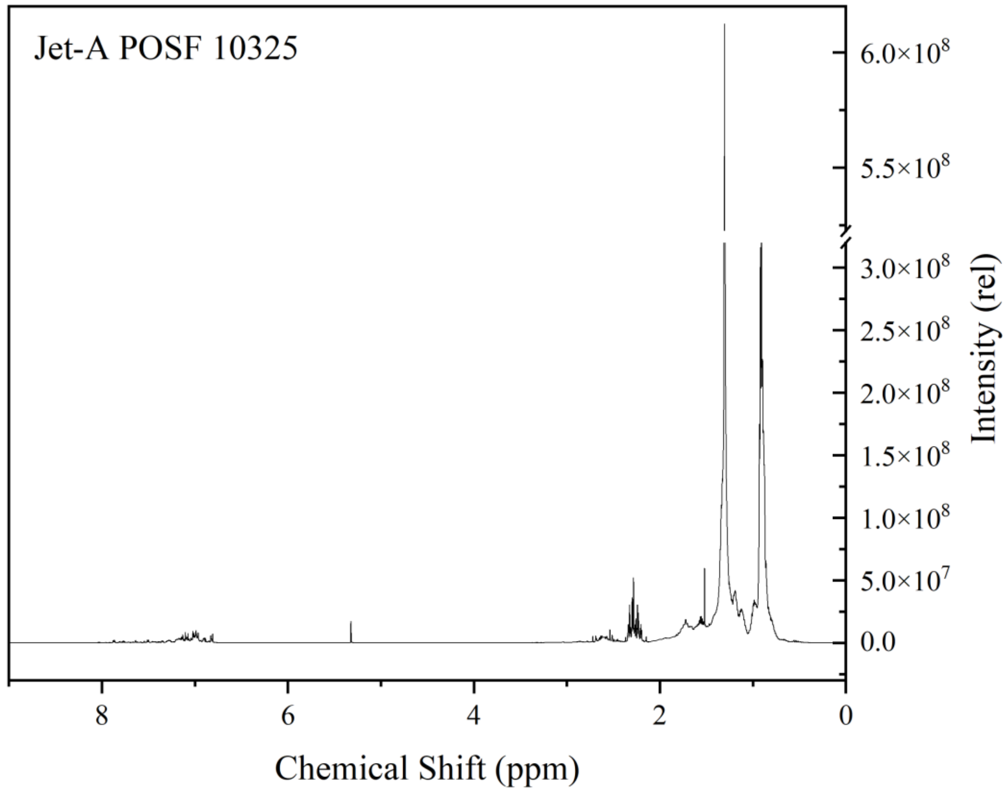

Figure S1-1. ^{1}H NMR spectrum of Jet-A POSF 10325 with an axis break to allow for the intensities of the aromatic region (+7 ppm) to be visible.

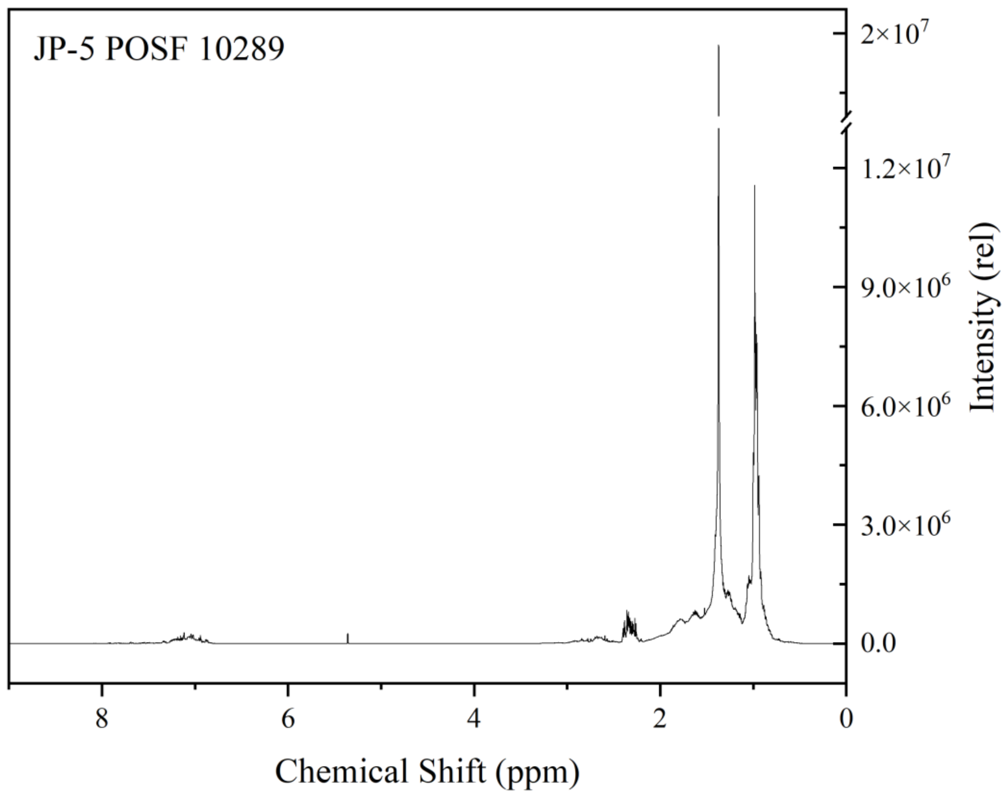

Figure S1-2. ^{1}H NMR spectrum of JP-5 POSF 10289 with an axis break to allow for the intensities of the aromatic region (+7 ppm) to be visible.

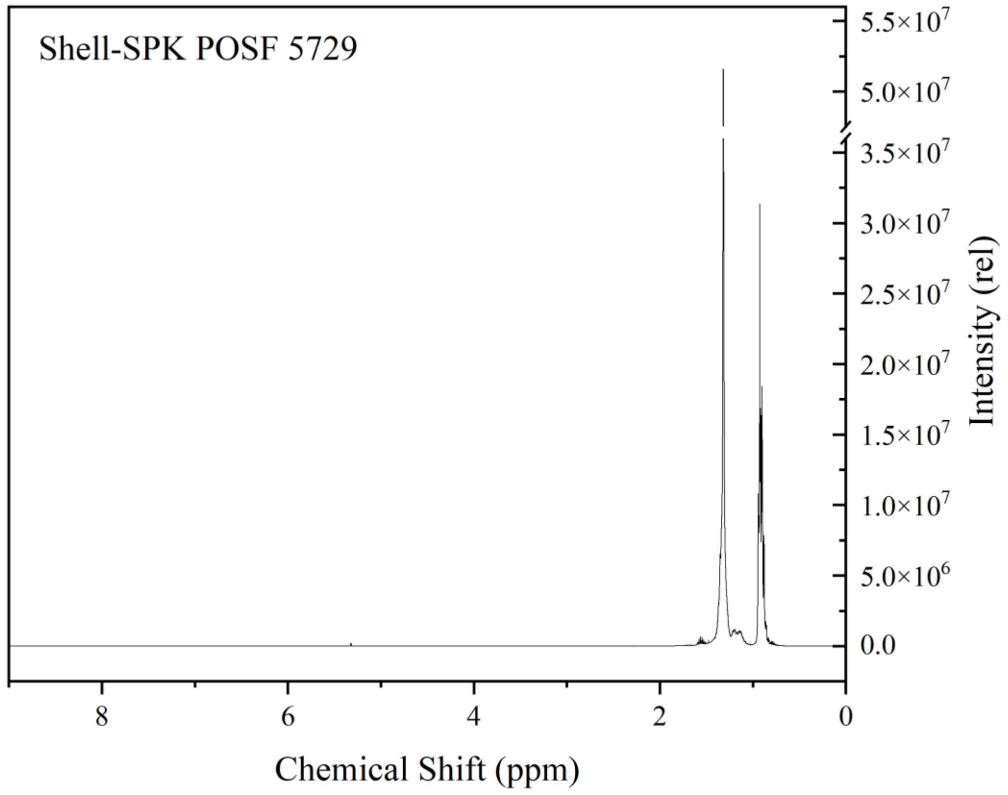

Figure S1-3. ^{1}H NMR spectrum of Shell-SPK POSF 5729 with an axis break to allow for the intensities of the lower paraffinic peaks (<2 ppm) to be visible.

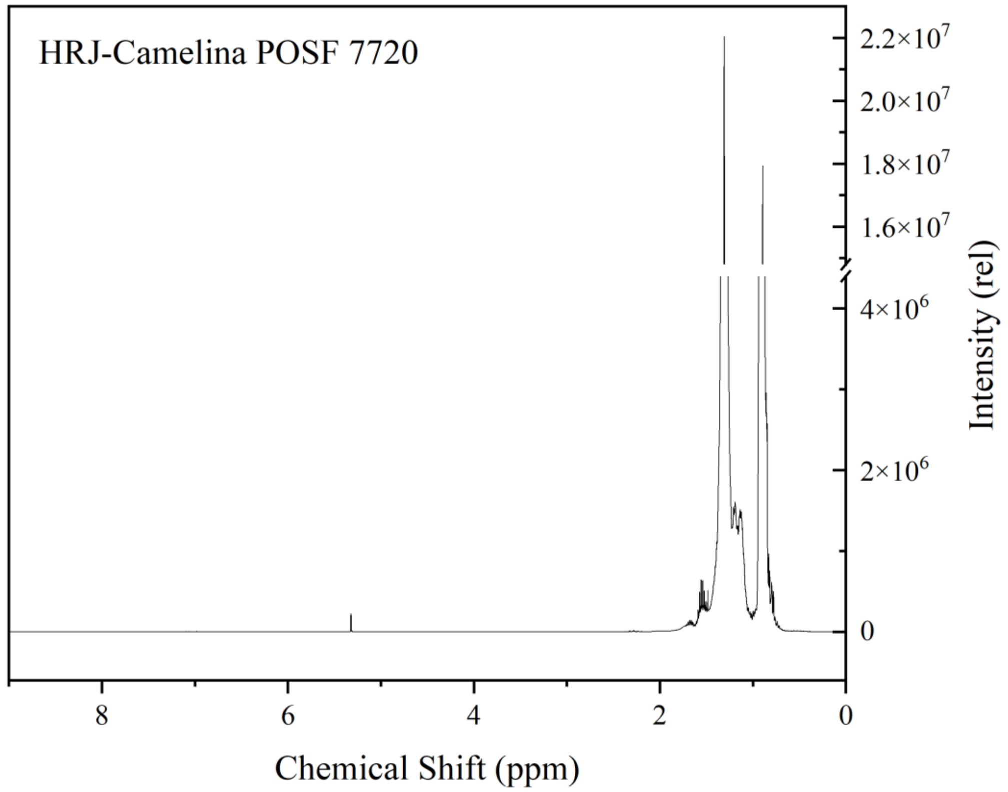

Figure S1-4. ^{1}H NMR spectrum of HRJ-Camelina POSF 7720 with an axis break to allow for the shapes of the full paraffinic peaks to be visible.

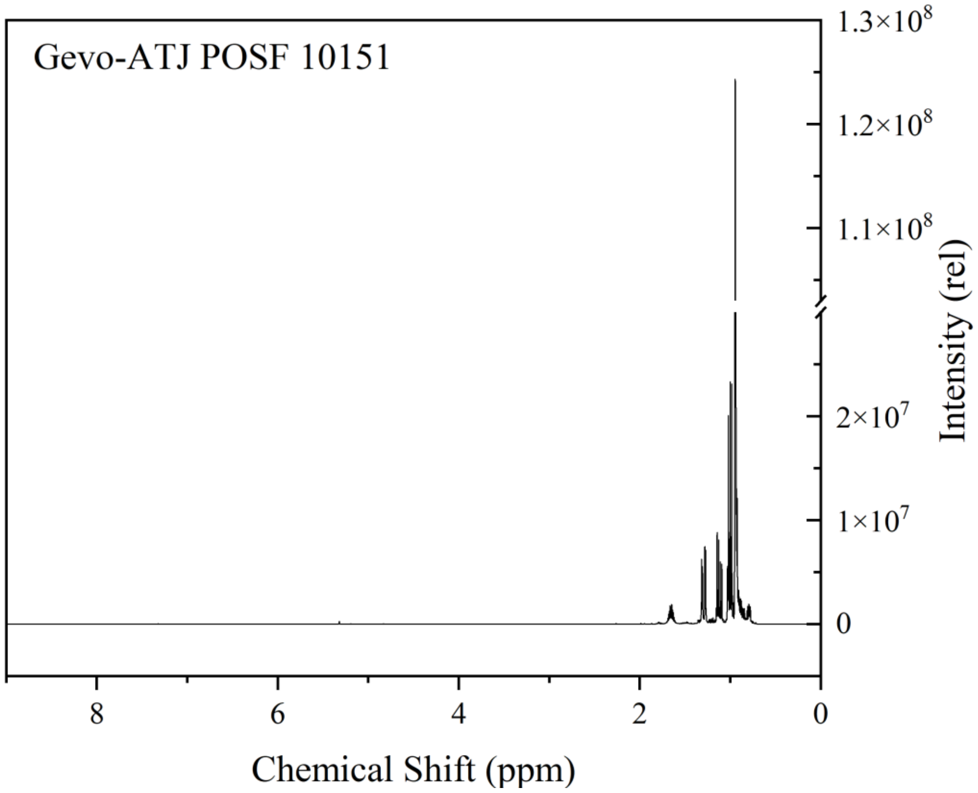

Figure S1-5. ^{1}H NMR spectrum of Gevo-ATJ POSF 10151 with an axis break to allow for the intensities of the <2 ppm to be visible.

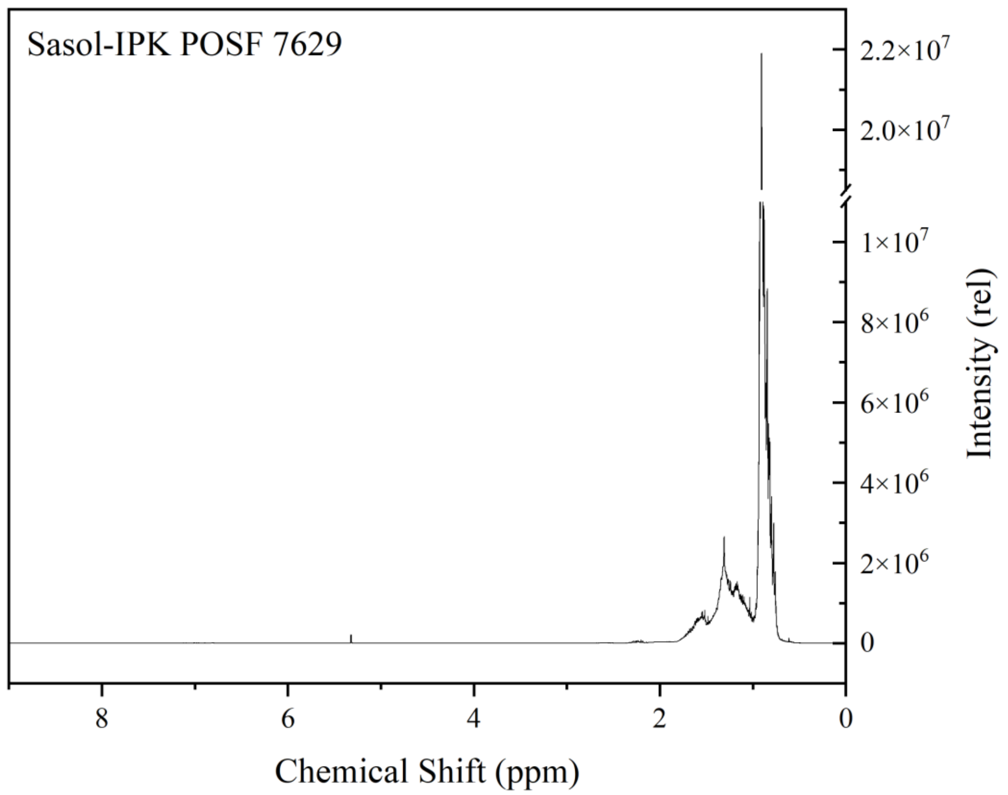

Figure S1-6. **^{1}H NMR spectrum of Sasol-IPK POSF 7629 with an axis break to allow for the intensities of the aromatic region (7+ppm) to be visible.**

S2 – Temperature distribution of density and surface tension datasets:

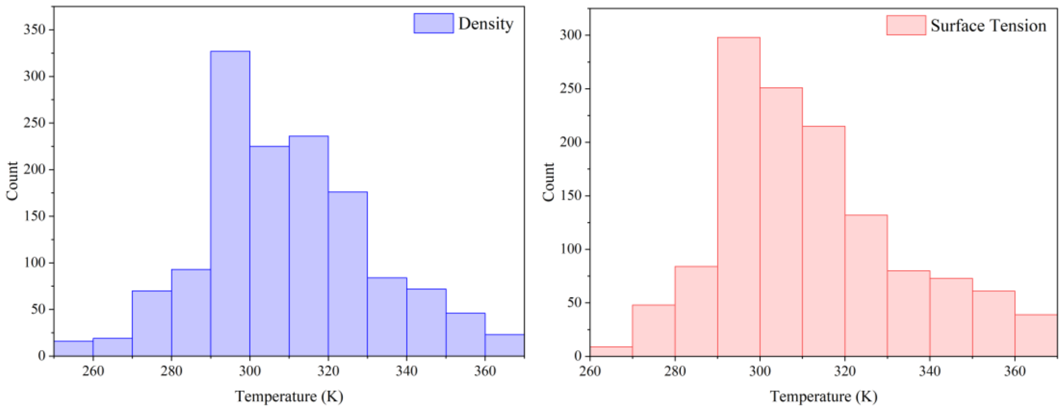

Figure S2-1. Temperature histograms for density and surface tension training data. This plot shows how the temperature is distributed for each of the datasets. The centralized peaks around 300K are due to large ambient temperature measurements.

S3 – Experimental data for surface tension and liquid density:

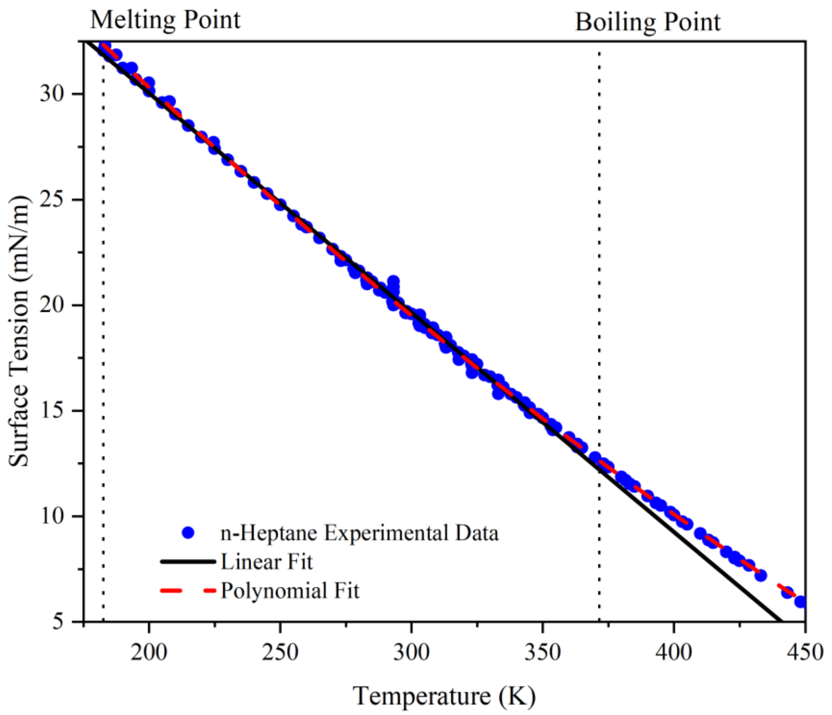

Figure S3-1. Surface tension temperature-dependence of n-heptane over a large range of temperature. This shows that until we near the phase boundary the surface tension temperature dependence can assumed to be linear. n-Heptane was chosen as an extremal example here as most components in jet fuel are higher in carbon number than C7, which means they have higher boiling points and therefore less deviation from linearity at the highest relevant temperatures. The data presented in the figure is from NIST Thermodynamic Data for Pure Compounds.[1]

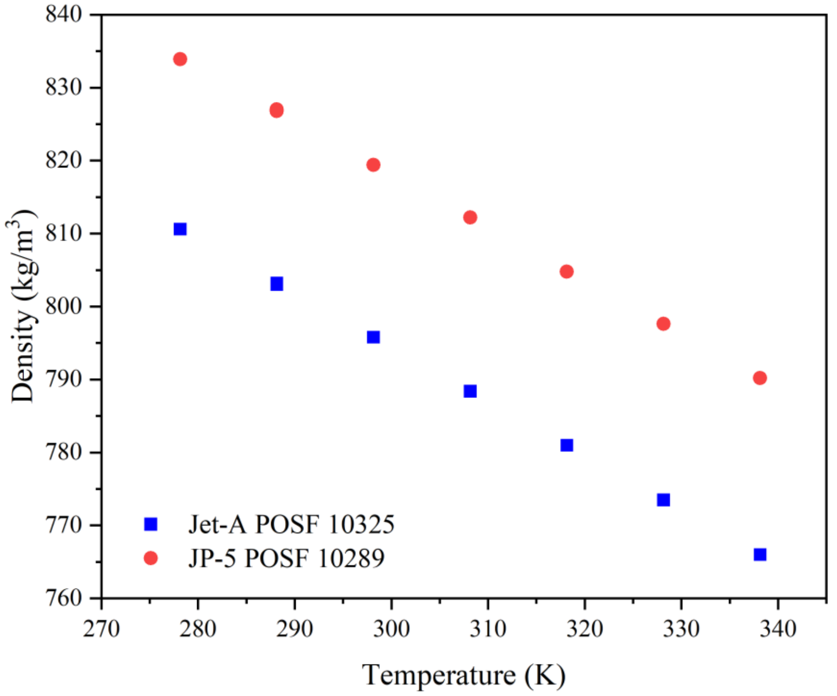

Figure S3-3. Experimental density temperature-dependence of Jet-A POSF 10325 and JP-5 POSF 10289.[2] This plot shows that the temperature dependences of fuel density for these real jet fuels

is essentially linear over the temperature range considered. The data present in the figure is from Edwards et al. [2, 3]

S4 - Parity Plots for Liquid Density and Surface Tension Separate Analysis for Training and Testing Datasets:

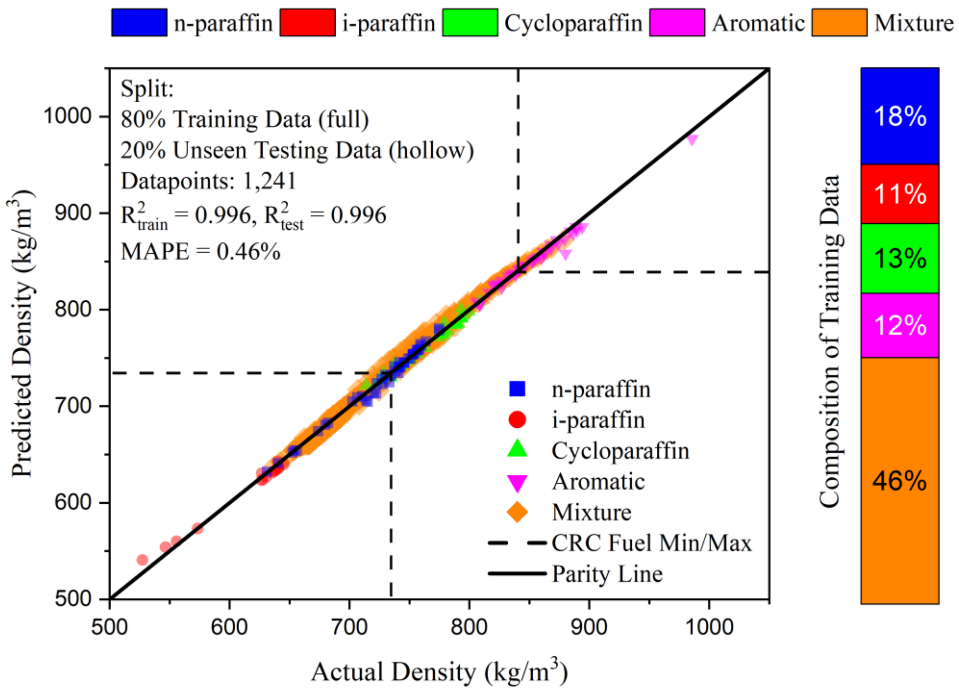

Figure S4-1: Training data Parity Plot for Temperature-Dependent Liquid Density.

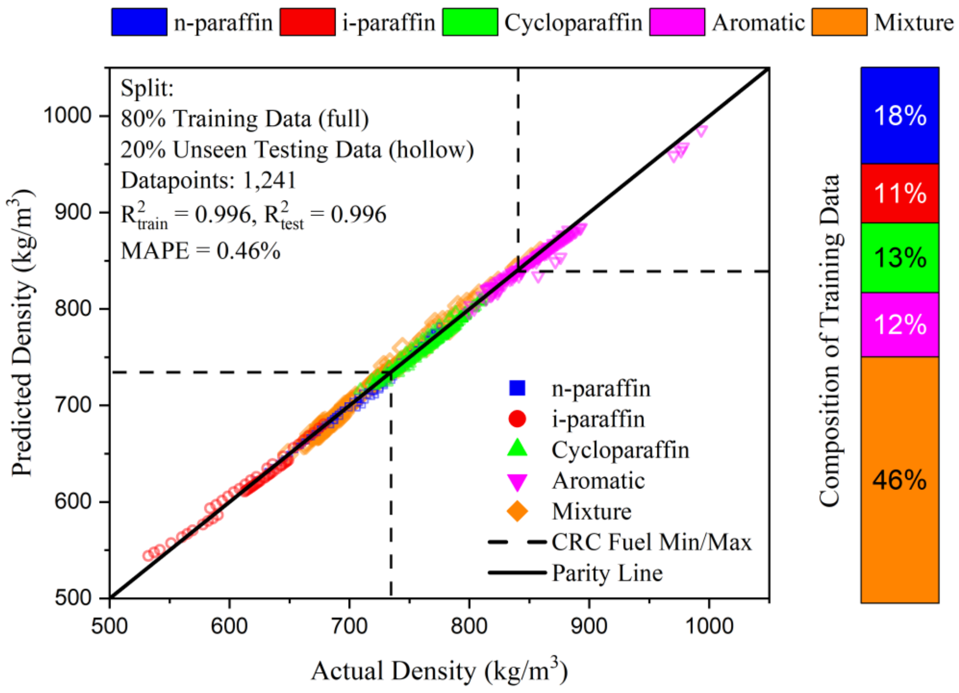

Figure S4-2: Testing data Parity Plot for Temperature-Dependent Liquid Density.

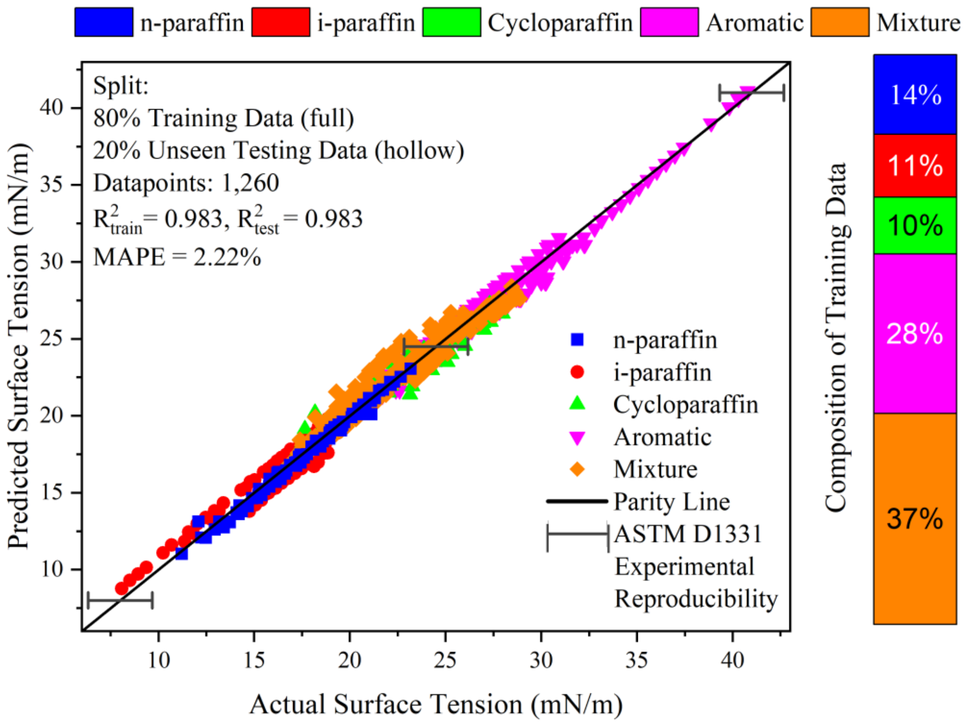

Figure S4-3: Training data Parity Plot for Temperature-Dependent Surface Tension.

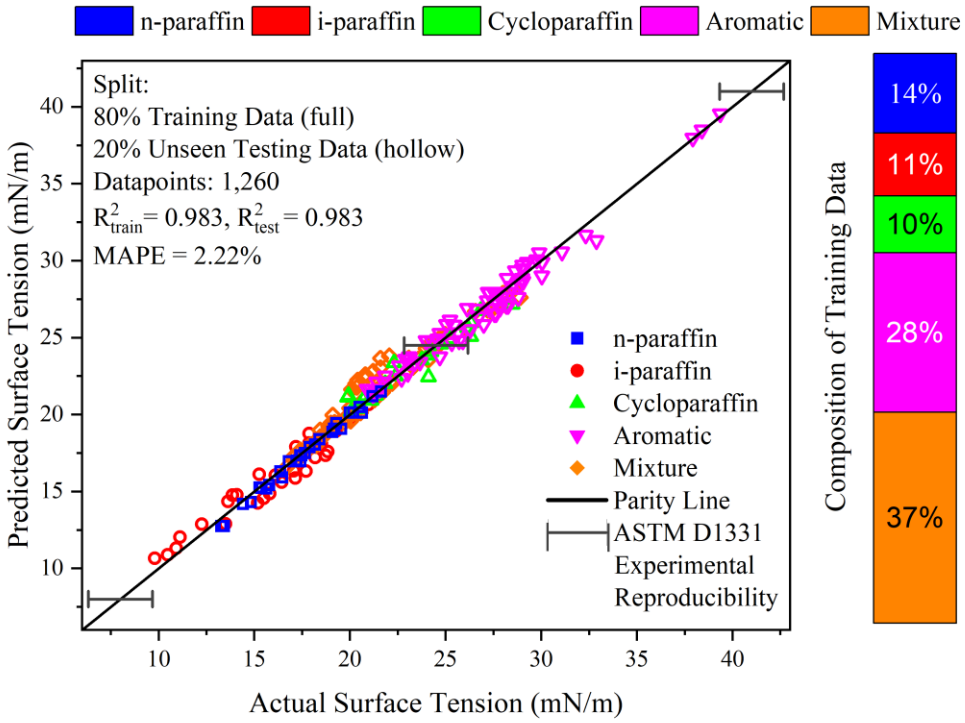

Figure S4-4: Testing Parity Plot for Temperature-Dependent Surface Tension.

References

(1) Diky, V.; Muzny, C. D.; Smolyanitsky, A. Y.; Bazyleva, A.; Chirico, R. D.; Magee, J. W.; Paulechka, Y.; Kazakov, A. F.; Townsend, S. A.; Lemmon, E. W.; Frenkel, M. D.; Kroenlein, K. G. *NIST Standard Reference Database 103b: Thermodynamic Data for Pure Compounds*; NIST/Thermodynamics Research Center (TRC). Retrieved from https://app.knovel.com/hotlink/toc/id:kpLTI00007/nist-standard-reference/nist-standard-reference (accessed 2024-08-01).

(2) Edwards, J. T. *Jet Fuel Properties*; Air Force Research Laboratory: Wright-Patterson AFB, OH, 2020.
(3) Edwards, J. T. *Reference Jet Fuels for Combustion Testing*. Presented at the 55th AIAA Aerospace Sciences Meeting, Grapevine, TX, January 9–13, 2017.
